# Factors Affecting the Expression of Recombinant Protein and Improvement Strategies in Chinese Hamster Ovary Cells

**DOI:** 10.3389/fbioe.2022.880155

**Published:** 2022-07-04

**Authors:** Zheng-Mei Li, Zhen-Lin Fan, Xiao-Yin Wang, Tian-Yun Wang

**Affiliations:** ^1^ School of Life Science and Technology, Xinxiang Medical University, Xinxiang, China; ^2^ International Joint Research Laboratory for Recombinant Pharmaceutical Protein Expression System of Henan, Xinxiang, China; ^3^ Institutes of Health Central Plain, Xinxiang Medical University, Xinxiang, China; ^4^ Department of Biochemistry and Molecular Biology, Xinxiang Medical University, Xinxiang, China

**Keywords:** recombinant protein, CHO, difficult to express, expression vector, recombinant therapeutic proteins

## Abstract

Recombinant therapeutic proteins (RTPs) are important parts of biopharmaceuticals. Chinese hamster ovary cells (CHO) have become the main cell hosts for the production of most RTPs approved for marketing because of their high-density suspension growth characteristics, and similar human post-translational modification patterns et al. In recent years, many studies have been performed on CHO cell expression systems, and the yields and quality of recombinant protein expression have been greatly improved. However, the expression levels of some proteins are still low or even difficult-to express in CHO cells. It is urgent further to increase the yields and to express successfully the “difficult-to express” protein in CHO cells. The process of recombinant protein expression of is a complex, involving multiple steps such as transcription, translation, folding processing and secretion. In addition, the inherent characteristics of molecular will also affect the production of protein. Here, we reviewed the factors affecting the expression of recombinant protein and improvement strategies in CHO cells.

## Introduction

In recent years, the demand for biopharmaceuticals has been increasing and innovative and improved bioprocessing technologies are essential to meet the demand for industrial production of products. As an important part of biological drugs, recombinant therapeutic proteins (RTPs), especially RTPs represented by recombinant antibodies, have achieved good efficacy and great economic benefits and have received more and more attention. Mammalian cells have become the main host cells for the production of RTPs because of their ability to perform post-translational modifications (PTMs) such as correct folding and glycosylation of recombinant proteins. Since the production of tissue type plasminogen activator (tPA) in Chinese hamster ovary (CHO) cells in 1986, CHO cells have become the production host of most approved recombinant proteins and recombinant antibody drugs due to their high density suspension growth, PTMs similar to humanized cells, easy gene operation and process scale-up. This expression system has been used to produce 84% of the antibodies approved during 2015–2018, accounting for more than half of all approved antibodies during this period (Walsh, 2018). By 2019, all six of the top ten best-selling drugs were produced in CHO cells (Urquhart, 2020). RTPs are the fastest growing part of the biopharmaceutical industry, with the US Food and Drug Administration (FDA) approving an average of more than 25 new approvals per year from 2014 to 2020. It is predicted that the global biopharmaceutical market value will reach $389 million by 2024 (O'Flaherty et al., 2020).

With the advancement of CHO cell line development and process optimization, the yields of recombinant mAbs (monoclonal antibodies, mAbs) of CHO cells have achieved as high as 5 g/L, or even more than 10 g/L (Alves and Dobrowsky, 2017; Handlogten et al., 2018). Although CHO cell platforms have been widely used to produce RTPs, there are still inherent limitations in the synthesis and secretion of many complex RTPs, such as low productivity, growth restriction and expression instability, low resistance to culture-related stresses and high costs of production. The reasons of those are that intracellular protein processing can be affected by many factors, such as cellular secretory capacity, protein aggregation or degradation, and protein folding (Kaneyoshi et al., 2019), which results in unsatisfactory expression or difficult-to-express of RTSs. It has become urgent challenges to express specific protein in specific cell line with good quantity and quality using strategies involving genetic modification, optimization of expression vector, cell line modification and process optimization.

## Recombinant Protein Expression Process in Chinese Hamster Ovary Cell

From gene structure to correctly folded protein products, a series of procedure are involved, such as transcription, translation, PTM, protein folding, and secretion et al. The nucleotide sequence of its coding region determines the amino acid sequence of the corresponding protein. Transcription process can control protein production in CHO cell, and the stability of mRNA is directly related to the number of translation products mRNA produced by transcription is prone to degradation, so the degradation rate of mRNA directly affects the expression level of protein (Narula et al., 2019). Mature mRNAs need to be processed into the translation process by capping, splicing and polyadenylation, and can also affect the translation process with the structure of the composition of (eukaryotic initiation factors eIFs) and ribosomal proteins, and mRNAs can be degraded by co-translation. Studies have found that codon selection can affect the rate of ribosome elongation, which in turn affects the rate of mRNA degradation (Bicknell and Ricci, 2017), Others have found that mRNA levels and expression levels are not consistent (Reinhart et al., 2014). After transcription in the nucleus, mRNA is transported to the cytoplasm and recognized and translated by ribosomes (Baek et al., 2017), and peptides of secreted proteins are translocated into the ER in a co-translational manner, followed by protein folding and maturation in the ER lumen tube, transport of correctly folded proteins into the Golgi apparatus for PTMs, and finally secretion into the extracellular environment in the form of secretory vesicles to function. Unfolded or terminally misfolded proteins can be removed by ER-associated protein degradation (ERAD) (Ruggiano et al., 2014; Wu and Rapoport, 2018; Mathias et al., 2020). Accumulation of unfolded or misfolded proteins in the ER lumen beyond the transport capacity impairs ER function, triggering the unfolded protein response (UPR) ultimately leading to apoptosis and affecting yield (Hetz, 2012; Hetz and Papa, 2018; Mathias et al., 2020).

When proteins are synthesized on ribosomes, signal recognition particle (SRP) in the cytoplasm recognizes signal peptides that newly generate 5∼30 amino acids at the N-terminus of proteins and function to transport proteins into the correct subcellular compartment (Saraogi and Shan, 2011). Next, SRP transfers SRP and the signal recognition particle-ribosome nascent chain (SRP-RNC) combinatorial complex to receptors on the ER membrane, which is finally transferred to the lumen of the ER, and then the signal peptide is cleaved by signal peptidase. Therefore, the transport of protein to the endoplasmic reticulum is the rate-limiting step of the secretory pathway, and the signal peptide sequence can promote the intracellular transport of recombinant protein products to secretory organelles and promote expression. If the nascent polypeptide chain is inefficiently translocated to the endoplasmic reticulum, SRP complex function may lead to miscleavage of the signal peptide, resulting in inefficient assembly and low expression of the antibody light chain (Reinhart et al., 2014).

Polypeptide chains with complete primary structure can have normal biological activity only when they fold into the correct three-dimensional spatial structure. If the folding process is wrong and a misplaced spatial structure is formed, they will lose their biological function and may even cause diseases. Typically correctly folded proteins form small vesicles that leave the ER, which move toward the Golgi and fuse with its membrane. Transport inside the Golgi and transport of proteins from the Golgi to the plasma membrane are also mediated by vesicles. If the high concentration of heterologous protein exceeds the secretory capacity of CHO cells, it will lead to inefficient secretion, protein degradation, intracellular and extracellular aggregation, etc., which in turn affects the yield of protein (Delic et al., 2014). The inherent properties of other molecules may also contribute to the susceptibility of proteins to degradation, aggregation, and other adverse protein-protein interactions that may lead to cytotoxicity. The cells themselves may produce toxicity, one is intracellular toxicity, involved in cellular regulation, and the other is extracellular toxicity, involved in negative feedback regulation. Usually most polypeptide chains can fold correctly, but a small proportion of proteins are still difficult to fold correctly.

PTMs, usually in two ways, through proteolytic cleavage and covalent modification through amino acids, may lead to changes in protein characteristics and function (Spahr et al., 2018; Bryan et al., 2021). When the protein itself is improperly designed or the DNA coding sequence is inappropriate, it will lead to the lack of PTM (Unzueta et al., 2015), insufficient supply of chaperones, difficulty in the formation of interchain and intra-chain disulfide bonds, and ineffective vesicle transport (Delic et al., 2014) or other adverse forms of protein-protein interactions, which in turn will affect the expression of proteins and affect the yield and quality of recombinant proteins.

## Overcoming Difficulty in Expressing Recombinant Protein in Chinese Hamster Ovary Cells and Improving Strategies

Although the technology of producing recombinant protein by CHO cells has been more mature and a large number of recombinant proteins have been successfully produced, there are still some proteins that are DTE and proteins with low expression levels. To solve this problem, the main strategies mainly include ar the increasing the cell viability, viable cell density, and cell specific production rate (Qp) through some molecular and cell methods, using genetically engineered cell lines.

## Genetic Modification

Ribosomes have traditionally been considered deciphers of the 64-word genetic code translated into complex proteins, while ribosomes can also shape the transcriptome by controlling mRNA stability. In recombinant protein production, the expression rate, yield, and final product quality are affected by the codon bias of the relevant gene and the codon bias of the expression system (Quax et al., 2015). Although all codons affect the translation rate, some codons will be rapidly deciphered, while others will cause ribosome pausing due to the relative cognate tRNA concentration. Due to the species preference of codons, codon optimization can be used to overcome the difficulty in protein expression or improve the expression level of recombinant proteins in recombinant protein expression (Ang et al., 2016; Chung et al., 2013). It is generally accepted that codon bias can improve translation efficiency by modulating the elongation rate of the process (Quax et al., 2015). Heterologous expression of recombinant human interferon beta (rhIFN-β) gene in suspension-adapted Chinese hamster ovary (CHO-s) cells with optimized codons to increase the GC content at the third position of each codon increased the expression level by 2.8-fold (Shayesteh et al., 2020). Overexpression of transcription factors such as ZFP-TF, ATF4, or GADD34 significantly increased the yield of various cellular recombinant proteins compared with parental cells, up to 10-fold (Kwon et al., 2006; Omasa et al., 2008). Due to the low frequency of availability of certain transport RNA (tRNA) molecules, codons associated with them are slowly translated. Codons with higher tRNA abundance will therefore translate more accurately (Shah and Gilchrist, 2010). Another key protein that plays an important role in protein biosynthesis is mammalian target of rapamycin (mTOR), which enhances the ectopic expression of mTOR gene and can also significantly improve the cell growth (increase cell size and protein content), proliferation (increase cell cycle progression), viability (decrease apoptosis), robustness (decreased sensitivity to suboptimal growth factors and oxygen supply), and specific productivity of secreted human glycoproteins of recombinant CHO cells. Culturing an mTOR transgenic CHO-derived cell line for the secretion of therapeutic immunoglobulins with an antibody of 50 pg/cell/day increased fourfold compared to the parental production cell line (Dreesen and Fussenegger, 2011).

Gene sequence optimization can also adjust the GC content of genes, avoid base duplications, and eliminat restriction enzyme recognition sites, and also avoid similarity to important RNA motifs located in open reading frames that may interfere with mRNA processing and translational function, because they may cause ribosome pausing, in addition to factors such as CpG content and TATA boxes. Another 5′ untranslated region (5′ UTR) insertion into the RNA hairpin structure, the “regulatory element (RGE)”, also improves expression (Eisenhut et al., 2020). In addition to gene sequence optimization, sequence motifs such as complementarity determining region three influences the rate of LC-HC dimerization during MAb synthesis, results in product-specific difference of yield (Pybus et al., 2014).

## Optimization of Expression Vector

In mammalian cells, a highly complex process controlled by multiple DNA signaling elements and corresponding trans-acting factors is called transcription. In this cascade, promoters play a key role in integration and transcriptional signal processing, therefore, selecting the correct promoter, optimizing the combination of the promoter with different regulatory elements, and preventing promoter methylation can improve the expression and stability of recombinant proteins (Kim et al., 2011; Ho et al., 2015). In CHO cells, human cytomegalovirus immediate-early enhancer/promoter (hCMVp), simian virus 40 early promoter (SV40E), CMV enhancer/chicken β-actin promoter (CAG), human elongation-1α promoter (HEF-1α) and Chinese hamster elongation factor-1α promoter (CHEF-1α) are commonly used to express GOI, and CMV promoter has the highest transgene expression level and the highest transfection efficiency, and SV40 promoter can maintain transgene expression more stably during long-term culture (Wang et al., 2016). Generally, selectable marker genes are driven by weak promoters (Nguyen et al., 2019), hypomethylation of DNA in promoter regions can improve gene transcriptional activity, and acetylation of acetylated histones (Dong et al., 2019) can also play a role in the transcriptional control of active genes.

Due to the structure of heavy chain (HC) and light chain (LC), the matching of HC and LC is need to be considered in the construction of expression vector. When free LC does not bind to HC, HC is misfolded, assembled and accumulated. LC peptides are very important for the efficient folding and assembly of Mabs. IgG antibody sequences with high aggregation/aggregation tendency are more likely to form Russell bodies in the ER lumen. LC and HC cannot be completely and correctly folded and assemble to form heterodimers in the endoplasmic reticulum and Golgi apparatus, which will cause inefficient assembly and affect antibody yield (Kaneyoshi et al., 2019) and overall Qp of cells (Ishii et al., 2014), so the LC expression level is high and the secretion rate is high. Compared with HC, LC excess is conducive to antibody production, and the ratio of HC to LC can be optimized by optimizing the number of transfected DNA or vector design. If a two-plasmid system is utilized, the HC and LC are located on different vectors and different plasmid ratios are used for transfection. In addition, single-plasmid systems can also be used to control HC to LC expression with different ratios using IRES (internal ribosome entry site, IRES) mediated vectors up to 1.15 g/L, double or tricistronic vectors (Li et al., 2020). The polycistronic vector contains IRES and Furin-2A (F2A), and the expression of multiple cistrons on a single vector is achieved by IRES and F2A (Ho et al., 2013) to optimize the expression of recombinant antibodies.

It has been found that random transgene insertions may be associated with position effects. Inefficient transgene expression is mainly due to transcriptional silencing, involving chromatin condensation, histone deacetylation and methylation at CpG sequences. By changing the structure of chromatin through specific chromosomal elements and maintaining chromatin in an open state, the transcription level of target genes can be increased. For example, ubiquitous chromatin opening element (UCOE) (Neville et al., 2017), as well as matrix attachment region (MAR), upstream chromatin opening element and locus control region, can prevent transgene silencing and are used to improve transgene expression (Li et al., 2018). MARs are DNA sequences that can interact with the nuclear matrix in eukaryotic genomes. Through the binding of MARs to the nuclear matrix, chromatin can form independent ring structures that regulate the transcription and expression of genes and reduce transgene silencing due to position effects. It has been found that MARs increase transgene expression levels in mammalian cell expression systems and reduce differences in expression levels between transgenic individuals, thereby inhibiting transgene silencing (Chang et al., 2014). MARs can also improve transgene expression levels in CHO cells, and there is a distance effect in MAR-regulated transgene expression (Zhang et al., 2014; Zhang et al., 2020).

## Cell Line Modification

The entire “CHO cell system” includes a variety of different cell lines, which are likely to come from the first isolation of clone, naturally immortalized CHO in 1956 (Puck et al., 1958). The productivity of dihydrofolate reductase (DHFR) deficient CHO cell can increase with gene copy number amplification (Yeo et al., 2017), which is commonly used for RTPs production.

In general, the factors that affect product quality depend on the type of RTPs and the rCHO cell line used. In a mathematical modeling of recombinant Mab manufacture in seven GS-CHO cell lines, the improving of Mab production through cell line modification should be cell line specific (O'Callaghan et al., 2010). The difference in integration site of GOI might influence the production of Mab in the strategies of cell line modification. Efficient expression of the transgene during development of an rCHO cell line based on random integration of the transgene is highly dependent on the number of gene copies integrated into the genome as well as the location of the integration. The latter is greatly influenced by changes in position effects, which are influenced by local permeability of the site and proximity and interaction with local and distal enhancers. It has been shown that transgenes with high copy number are not always consistent with high cell productivity (Reisinger et al., 2008), but the number of integrated transgenes will affect the expression of certain cell lines (Chusainow et al., 2009). Integration sites in different structural genomes and regulatory genomes as well as vector regulatory elements and their structural rearrangements can lead to clonal variation (Grav et al., 2018). Site specific integration has been used for CHO cell line development and engineering (Igarashi and Kashiwagi, 2021), the site is important for the production of GOI.

**FIGURE 1:**
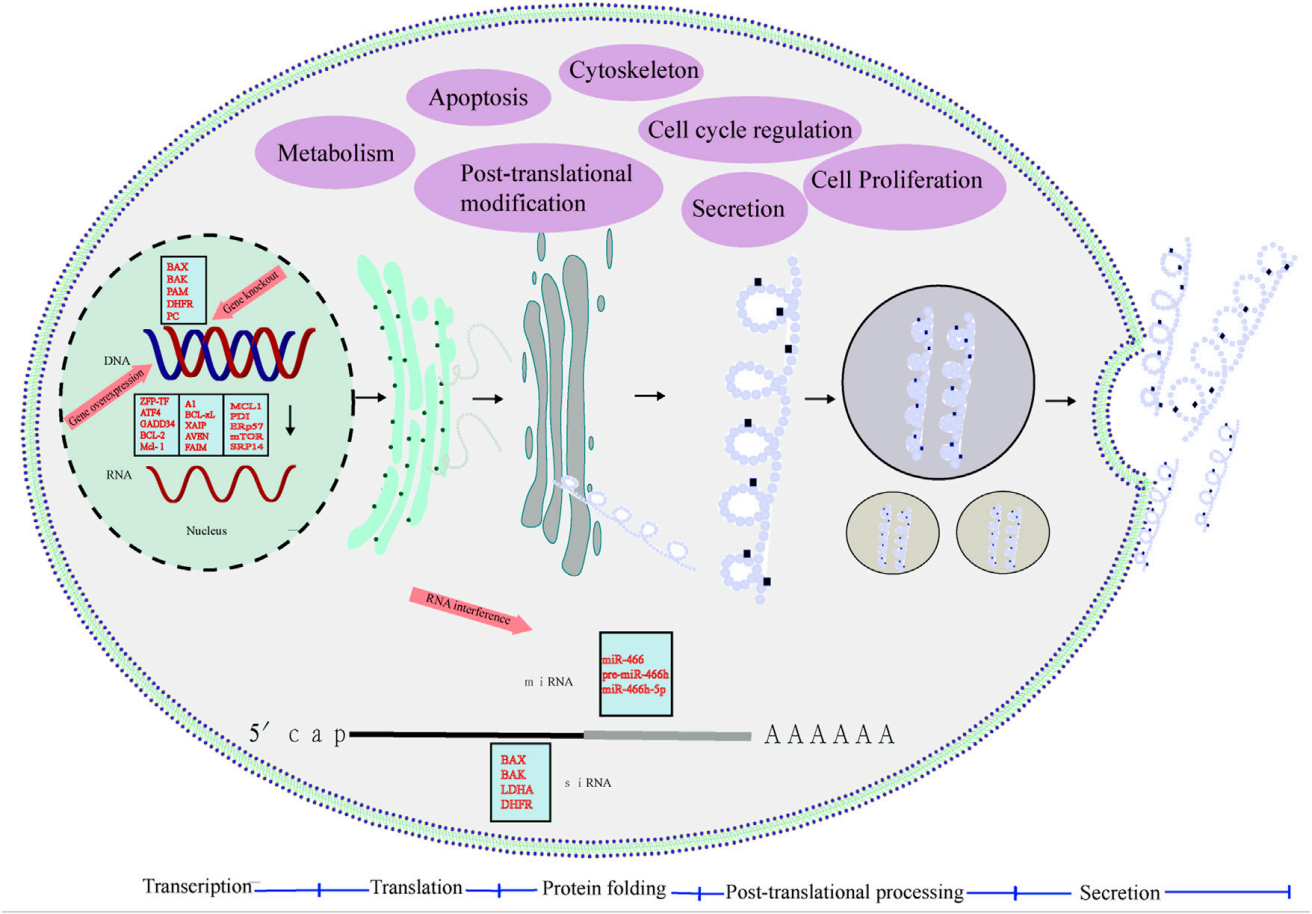
Process of protein synthesis. mRNA is transcribed in the nucleus, transported to the cytoplasm and translated by the ribosome, and then the protein binds to the rough endoplasmic reticulum, folding and processing before entering the Golgi apparatus. The soluble protein is PTMs and then secreted through the secretory pathway. Through cell engineering strategies such as anti-apoptotic engineering, metabolic engineering, cell cycle engineering, and protein PTMs engineering, overexpression of dominant genes, gene knockout, and siRNA interference inhibit unfavorable gene expression, transform mammalian cell lines, and improve cell lines Performance, effectively improve cell viability and increase the production of recombinant protein.

It is crucial to randomly integrate the transgene into CHO host cells and select the appropriate cell line to produce the protein of interest (POI). Generally, the most commonly used methods to engineer cell lines include gene overexpression and gene knockout, and the former method is to introduce the GOI into the host cell to realize the overexpression of POI. Knockout is more specific to delete a gene from the genome by chemically and radiation-induced random mutation or gene editing directed mutation, or to shut down its function and target genetic engineering, which is superior to random mutation. In this regard, the most advanced technologies mainly include zinc finger nucleases (ZFNs), transcription activator-like effector nuclease transcription activator-like effector nuclease (TALENs) and regularly clustered regularly interspaced short palindromic repeats (CRISPR)/CRISPR-related protein 9 (Cas9) systems (Dong et al., 2019; Carver et al., 2020), which are able to delete or introduce proteins in CHO cells while improving the productivity of recombinant proteins, which in turn promotes product quality (Grav et al., 2018). In addition, the production of rCHO cell lines is allowed by multi-copy number targeted integration to support the horizontal production of RTPs by their industrial production (Sergeeva and Lee, 2020) (Figure 1).

Mammalian cell lines are engineered by cell engineering strategies, such as anti-apoptotic engineering, metabolic engineering, cell cycle engineering, and protein PTM engineering, overexpression of dominant genes, gene knockout, and siRNA interference to inhibit unfavorable gene expression to improve the performance of cell lines, effectively improve cell viability, and improve the yield of recombinant proteins. In addition, non-coding RNAs technology can also improve and improve cell lines in growth, apoptosis inhibition, metabolism, yield, and the ability to express glycosylated recombinant proteins (Table 1), with great potential (Raab et al., 2021).

**TABLE 1:** Overcoming the difficulty in expressing recombinant protein in CHO cells and improving cell line modification strategies.

Cell engineering technology	Strategy	Targeted gene	Engineered phenotype	References
Antiapoptotic cell engineering	Increased expression of anti-apoptotic genes	Bcl-2 family (Bal-2, Mcl-1, Mcl-XL, A1, X-linked inhibitor of apoptosis protein (XIAP), apoptosis asparaginase inhibitor (AVEN), Fas apoptosis pathway inhibitor molecule (FAIM), myeloma cell leukemia 1 (MCL1)	Inhibition of nutrient starvation and accumulation of metabolic by-products in late culture	Sauerwald et al. (2002), Figueroa et al. (2004), Wong et al. (2006), Majors et al. (2009), Baek et al. (2017)
	Knockdown of apoptotic genes	Complete knockdown of Bcl-2-association-X protein (BAX) and Bcl-2 antagonist (BAK)	Inhibition of caspase activity (apoptosis core enzyme) enhances anti-apoptotic ability	Lim et al. (2006)
	Knockdown of pro-apoptotic genes	Caspase-3 and caspase-7	Improved viability and extended culture time	Sung et al. (2007)
	miRNAs regulate apoptosis	miR-466, pre-miR-466h, miR-466h-5p	Prolong the culture cycle and increase the density of viable cells	Druz et al. (2011), Druz et al. (2012), Druz et al. (2013)
Metabolic engineering	Improved overexpression of cellular metabolic genes	Vitreoscilla hemoglobin (VHb)	Change cell metabolic activity and prolong culture time and yield	Fussenegger and Bailey, (1998)
	Knockdown of key metabolic enzymes	Pyruvate carboxylase (PC)	Decreased lactate production and increased cell viability	Kim and Lee (2007)
Cytoskeleton and cell cycle engineering	Backbone key regulatory protein knockdown	Cofilin	65% (SEAP) and 47% (tPA) (more efficient production)	Hammond and Lee, (2012)
Protein PTMs engineering	Glycosylation site knockout	GDP-fucose (SLC35C1) and CMP-sialic acid (SLC35A1)	IgG producing strongly enhanced ADC	Zhang et al. (2012), Haryadi et al. (2013)
Protein synthesis engineering	Simultaneous overexpression of protein	Calnexin (CNX) and Calreticulin (CRT)	Specific thrombopoietin (TPO) productivity was increased 1.9-fold without affecting the cell growth and biological activity of recombinant TPO negatively affecting	Chung et al. (2004)

Cell apoptosis refers to the autonomous and orderly death of cells controlled by genes to maintain the stability of the internal environment, which can reduce apoptosis and prolong culture time during bioprocessing, thereby increasing volume product yield (Henry et al., 2020). Overexpression of anti-apoptotic genes can facilitate genetic engineering of CHO-producing cells. Apoptotic proteins include Bc1-2 family (Bcl-2, Mcl- 1, Bcl-XL, A1), X-linked inhibitor of apoptosis protein (XIAP), inhibitor of apoptosis asparaginase (AVEN), Fas apoptotic pathway inhibitor molecule (FAIM), myeloma cell leukemia 1 (MCL1), etc., which inhibit nutrient loss and metabolic byproduct accumulation in the late stage of culture, induce the occurrence of apoptosis and then prolong the culture time (Figueroa et al., 2004; Wong et al., 2006; Majors et al., 2009; Raab et al., 2021), and another strategy is to knock down bcl-2 associated X protein (BAX) and bcl-2 antagonist/killer (BAK) in weakly promote CHO cells and prolong the culture time of CHO cells in nutrient-poor or high-osmolarity media (Lim et al., 2006).

MicroRNAs are defined as small molecules of 22–25 nucleotides that can regulate approximately 100 mRNA targets involved in one or more regulatory networks simultaneously. Studies have demonstrated that up-regulation or down-regulation of microRNAs in host cells can change productivity levels by regulating cellular processes, such as apoptosis, cell cycle, histone methylation, and cell growth (Klanert et al., 2016; Muluhngwi et al., 2017; Amadi et al., 2020) and other non-coding RNAs technologies can also enable optimization of cell lines. Reduced expression levels of miR-23 in CHO cells can triple the specific expression level of secreted proteins from this cell without affecting cell growth (Kelly et al., 2015). In CHO cells, small interfering RNA (siRNA) is widely used for specific gene silencing to improve apoptosis resistance, glycosylation, metabolism, or specific productivity (Sarkar and Tran, 2021). Using siRNA pairs of caspase-3 (CASP3) and -7 (CASP7), both genes were silenced simultaneously, resulting in improved performance to 5 g/L in CHO cells treated with sodium butyrate (NaBu, a non-specific inhibitor of histone deacetylases (HDACs) (Sung et al., 2007). It has been found that miRNAs induce apoptosis in CHO cells in nutrient-poor medium, in which miR-466h induces apoptosis in CHO cells (Druz et al., 2011; Druz et al., 2012). Pro-apoptotic knockdown of pre-miR-466h in recombinant CHO-SEAP cells mediated by short hairpin RNA (shRNA) prolonged culture for more than 35 h and increased viable cell density by more than 40%. The above results demonstrate the potential of this novel approach to create more production cell lines by stably manipulating specific miRNA expression. Knockdown of anti-apoptotic genes (BCL2L2, DAD1, BIRC6, STAT5A, and SMO) by pre-miR-466h was up-regulated 2.1- to 12.5-fold, demonstrating that these genes are direct targets of miR-466h-5p in CHO cells (Druz et al., 2013) and directly affect protein yield.

Most metabolic engineering can reduce the accumulation of toxic by-products and improve protein production, which can regulate many biological process-related traits through CHO cell metabolic engineering, such as glycosylation (Richelle and Lewis, 2017). Changing of the metabolic activity of CHO by overexpressing specific genes can affect cell metabolism, and prolong the culture time and yield. Enhanced expression of Vitreoscilla hemoglobin (VHb) in CHO cells has been shown to increase tPA production 40%–100% (Fussenegger and Bailey, 1998) (Fussenegger and Bailey, 1998). During cell culture, the culture medium is gradually acidified with the accumulation of lactate, and lactate dehydrogenase (LDH) converts pyruvate to produce lactate. The decreasing in pH resulting from the increase in lactic acid ultimately resulted in the inhibition of cell growth. It can inhibit LDH by avoiding the oxidation of pyruvate to lactate, reduce lactate dehydrogenase A (LDHA) to enzyme activity, and down-regulate lactate levels without affecting cell proliferation and yield (Zhou et al., 2011). In addition, the simultaneous decreasing in LDH and pyruvate dehydrogenase kinase (PDHK) activities can lead to a decrease in lactate concentration and increase the antibody yield per unit volume (Fu et al., 2016).

Alterations in cytoskeletal protein expression are thought to affect cellular processes associated with recombinant protein production, including transcription, cell cycle progression, metabolism, and secretory vesicle trafficking. Differential expression of cytoskeletal proteins has been observed in modified or gene-amplified cell lines (Kuystermans et al., 2010), and both small interfering RNA (siRNA) and shRNA can increase the Qp of recombinant CHO cell lines (Hammond and Lee, 2012). Cell cycle detection key kinases control cell cycle and proliferation, and overexpression of cell cycle inhibitors such as p21cip1 or p27Kip1 leads to cell cycle arrest, but can increase cell Qp in CHO cells (Astley and Al-Rubeai, 2008).

## Optimization of Cell Culture Process

The substances and energy required for biochemical reactions such as new cell synthesis and cell metabolism are obtained from the culture medium. It was found that amino acids as the backbone in the medium component had a significant effect on product formation and critical quality attributes of mAbs. Recombinant protein production requires high amino acids to support not only host cell growth and heterologous recombinant protein synthesis, but also provide raw materials as carbon and nitrogen sources for nucleotide, lipid, ATP, and NADPH synthesis (Duarte et al., 2014). Nutrients in the medium are necessary for the cell growth environment. Among these components, amino acids constitute the constituent recombinant proteins and native CHO proteins, accounting for approximately 70% of the stem cell mass (Carrillo-Cocom et al., 2015). Mammalian cells can only produce some of the amino acids required for protein synthesis, while others are essential amino acids that need to be replenished from the outside. The consumption of extracellular amino acids has a negative impact on cell growth, culture time, specific productivity (qP), and monoclonal antibody glycosylation (Fomina-Yadlin et al., 2014). Amino acids are important sources of nitrogen and potential carbon for many intermediates in a large number of metabolic pathways when other sources are limited. Therefore, CHO cells must be supplemented with a sufficient number of essential amino acids to maintain continued survival, growth and proliferation, and with non-essential amino acids. In the process of cell proliferation, amino acids account for the majority of cell biomass consumption (Hosios et al., 2016). Studies have confirmed that post-translational modification of amino acid residues affects the diversity and quality of Mabs. Glutamine (Gln) has also been shown to restore or stimulate mTORC1 (mechanistic target of rapamycin complex 1) activity upon amino acid starvation (Tan et al., 2017) and can also affect protein yield through glutamine hydrolysis (Durán et al., 2012).

During the optimization of production process, the effects of culture medium on cell viability, metabolism, and product quality should be considered. In the early stage, necessary nutrients were provided by the serum of animal tissues or blood. Due to the regulatory limitations of mAb production, the serum-free medium formulation was invented, thus avoiding the use of serum in cell culture (Song et al., 2013). There are now many formulations available for different cell lines and different growth levels, including CD CHO (Gibco), Excell CD CHO (SAFC), etc., During culture, cells may experience a decrease in recombinant protein yield during prolonged culture, even during extension of the final bioreactor scale (Tharmalingam et al., 2018). Modern industrial culture of CHO cells must support high viable cell density, promote the synthesis and extracellular transport of biological products, and optimize nutrients and formulations of the medium is very important for protein quality and yield improvement (Ritacco et al., 2018).

Using CHO mAb cell line, the fed batch culture was increased by 12-fold compared with batch culture, while the optimal choline chloride to glucose ratio of batch culture CHO cells could be optimized, the culture time could be shortened, and the protein yield could be improved (Kuwae et al., 2018). It was found that during perfusion cell culture, the use of concentrated nutrient diet decreased the medium consumption by 1.8-fold, increased the volume expression by 1.67-fold, and also increased the cell density (Bielser et al., 2020). Chemical additives such as sodium butyrate (NaBu) and dimethyl sulfoxide (DMSO) can increase Qp in rCHO cells in a dose-dependent manner by inducing cell cycle arrest (Ha and Lee, 2014), decreasing oxidative stress (Ha et al., 2018), and inhibiting endoplasmic reticulum stress (Ha et al., 2019). However, these chemical additives are cytotoxic, can induce apoptosis, and have a negative impact on product quality in rCHO cell culture. Therefore, harmless and effective chemical additives are needed. Driven by high volumetric titers and a small footprint, perfusion-based bioprocess research has regained an interest from academia and industry. The future of perfusion bioprocesses is bright and that the fields of media optimization and continuous processing hold the greatest potential (Igarashi and Kashiwagi, 2021).

To better maintain product quality during culture, it is important to minimize the accumulation of host cell proteins in the culture medium, otherwise it will damage product quality. In the process of rCHO cell culture, there are thousands of host cell proteins (HCPs) in the culture supernatant, and the total number and number of different types of HCPs depend on the host cell type, culture method and culture time. Due to higher cell concentrations and longer culture cycles, the extracellular accumulation level of HCPs in fed batch cultures is much higher than that in batch cultures (Park et al., 2017). Removal of unnecessary HCPs by multiple secretome engineering of CHO cells can reduce the total amount and number of different types of HCPs in the culture medium (Kol et al., 2020). In addition, if specific HCPs that damage POIs are identified, the genes encoding these HCPs can be knocked out in CHO cells, thereby eliminating their accumulation in the culture medium. By using CRISPR/cas9-mediated methods, heavy sialylated glycoproteins such as EPO can be produced in batches by knocking out sialidase and pro-apoptotic genes in rCHO cells (Ha et al., 2020).

Cell culture parameters for Qp include partial pressure of carbon dioxide, osmolality, temperature, dissolved oxygen, and pH, and improvements in cell secretion, folding, and glycosylation are important ways to optimize mammalian cell production systems (Omasa et al., 2010). The optimal temperature for CHO cell growth is 37°C, and the temperature was controlled lower than 37°C during protein production (Sou et al., 2015; Torres et al., 2018), which can prolong the time of antibodies and other RTPs and improve the final product yields Long and low temperature two-phase cell culture process can improve product quality and purification yield (Gomez et al., 2018). pH value of culture medium is another important environmental parameter, can affect cell growth, cell metabolism, and the yield and quality of recombinant proteins in rCHO cells (Xie et al., 2016; Paul et al., 2019). CHO cells grow well at neutral pH (Michl and Park, 2019). The intracellular pH is not uniform, ranging from 4.7 to 8.0 in lysosomesand mitochondria (Jaworska et al., 2021). Every cellular process that occurs in intracellular organelles has an optimal pH, which shows optimal function when the pH value is reached. When the optimal pH deviates from a specific cellular process, it can cause changes in conformational enzymes and impair function. The osmotic pressure of the medium can also affect cell growth and recombinant protein production, and the osmotic pressure of CHO cells was 280∼320 mOsm. When osmolality deviates from this standard range, rCHO cells showed a decrease in Qp, but generally show an increase in Qp (Qin et al., 2019). High osmolality has a positive effect on the product quality of protein aggregation, but a negative effect on protein glycosylation. In addition, in rCHO cell culture, the parameters of CO_2_ and O_2_ dissolved oxygen should also be coordinated.

## Promote Correct Folding and Processing of Proteins

Molecular chaperones are a family of proteins whose role is to assist in the folding and targeting of proteins in normal and stressed cells. Insufficient supply of chaperones limits correct protein folding in recombinant protein production. Intramolecular chaperones can help protein folding and assist protein subunits to correctly fold with multiple subunits to assemble into intact proteins (O'Rourke et al., 2019) (Fatima et al., 2021). Following protein synthesis and translocation into the ER lumen, secreted proteins must be properly folded and modified with the help of chaperones and folding enzymes. Chaperone proteins in CHO cells can improve the productivity of difficult-to-express proteins, such as chaperone heat shock proteins (HSPs), which help protein folding, assembly, translocation, and degradation under cellular stress. It also prevents the aggregation of newly synthesized polypeptide chains into defective proteins. Protein disulfide isomerase (PDI) catalyzes the formation and cleavage of disulfide bonds and helps protein folding improve the productivity of Mabs (Borth et al., 2005), and its isomer ERp57 can increase protein yield up to 2.1-fold without reducing cell growth (Hwang et al., 2003). It should be pointed out that this would only be an effective strategy where this was the only limiting factor. Engineering of protein folding and assembly in the ER is inherently protein specific, and possibly Mab specific. Mabs, even of the same subclass, likely differ in their kinetics of folding and assembly and thus may benefit to a variable extent from overexpression of different ER molecular chaperones or foldases. PDI has a certain inhibitory effect on the formation of disulfide bridges in antibody LC. When PDI recognizes less antibody light chain (LC), the formation of disulfide bridges in antibody light chain (LC) is blocked, and secretion is blocked due to incorrect folding (Mathias et al., 2020). Paracycline B interacts with other proteins in the ER to form a partner complex that promotes protein folding. Chemical chaperones are compounds that improve ER folding capacity, promote protein folding in the ER, and enhance protein secretion. Expression levels were improved by optimizing chaperones, UPR transactivators, chemical chaperones, maximizing cell-specific yield and eliminating protein aggregation during transient expression (Johari et al., 2015). The introduction of promoted genes in protein folding, such as simultaneous overexpression of calnexin (CNX) and calreticulin (CRT), increased specific thrombopoietin (TPO) productivity 1.9-fold, did not affect the cell growth and biological activity of recombinant TPO, increased protein synthesis and secretion, and then increased the yield of recombinant protein in CHO cells (Chung et al., 2004).

Signal peptides transport proteins to the correct subcellular compartment, but natural signal peptides are not necessarily optimal (Kober et al., 2013). Moreover, signal peptides can be interchanged between different species (Knappskog et al., 2007). Therefore, the selection of appropriate signal peptides is conducive to the correct processing and secretion of proteins (Haryadi et al., 2015) Optimizing different signal peptides can improve recombinant protein expression (Cecconi et al., 2021).

## Toxic Protein Expression Strategy

Another factor causing insufficient protein production is the inherent toxicity of proteins. Avoiding exposure of cells to high concentrations of protein, or optimizing cell lines to resist toxic proteins, can promote growth and then increase yield. Protein toxicity can be mitigated by a variety of methods. One approach is to change the protein itself to reduce its cytotoxicity modify the protein to produce a more stable, less toxic form, and the other option is to change the promoter in the expression vector rather than the POI. The inherent characteristics of molecules can also lead to easy degradation, aggregation and other adverse protein-protein interactions of proteins, which leads to cytotoxicity. If the GOI is determined to be toxic, then one option to increase yield is to adapt host cells and reduce growth process by-products (Knappskog et al., 2007). Decreased sensitivity, protein toxicity, or degradation of cell lines cultured in the presence of low concentrations of protein can also be alleviated by reducing protein contact with production cultures. Avoiding the burden of recombinant protein metabolism during cell growth produces potential toxicity and inducible expression vectors can be applied (Ong et al., 2019). It can also be achieved by chemical supplementation, in which toxic proteins competitively inhibit interactions with cells through antagonists. Another approach is to use a perfusion growth system in which the culture supernatant is continuously removed by filtration and the cell mass is retained, which can improve cell density and volume productivity (Junne and Neubauer, 2018).

## Summary and Prospect

To improve the protein production capacity of recombinant proteins in CHO cells, the bottlenecks in transcription, translation, PTMs, protein folding and secretion during the production process must be recognized, and solutions must be developed for each rate-limiting step. Cell engineering technology can precisely control the relative expression of multiple functional gene components, which in turn affects protein yield. New therapeutic applications are opened up using new protein forms such as bispecific antibodies and fusion proteins. An understanding of the critical quality attributes of RTPs during CHO cell culture and their contributing factors is critical to the production of high quality RTPs. The proper assembly of these new RTPs may be more complex, which may affect productivity and potency. The solution strategy can start from the molecular design of proteins, combined with appropriate cell line, vector engineering and process optimization, based on the understanding of folding mechanism and the understanding of potential bottlenecks, optimize protein secretion and prevent aggregation, maximize the yield of recombinant proteins, reduce large-scale production costs, and promote the development of biopharmaceuticals.
